# Neu1 deficiency induces abnormal emotional behavior in zebrafish

**DOI:** 10.1038/s41598-021-92778-9

**Published:** 2021-06-29

**Authors:** Asami Ikeda, Mayu Komamizu, Akito Hayashi, Chiharu Yamasaki, Keiji Okada, Momoko Kawabe, Masaharu Komatsu, Kazuhiro Shiozaki

**Affiliations:** 1 The United Graduate School of Agricultural Sciences, Kagoshima University, Kagoshima, Japan; 2 Faculty of Fisheries, Kagoshima University, Kagoshima, Japan

**Keywords:** Animal behaviour, Glycobiology, Cognitive control, Psychiatric disorders

## Abstract

NEU1 sialidase hydrolyzes sialic acids from glycoconjugates in lysosomes. Deficiency of NEU1 causes sialidosis with symptoms including facial dysmorphism, bone dysplasia, and neurodegeneration. However, the effects of NEU1 deficiency on emotional activity have not been explored. Here, we conducted the behavioral analysis using Neu1-knockout zebrafish (Neu1-KO). Neu1-KO zebrafish showed normal swimming similar to wild-type zebrafish (WT), whereas shoaling was decreased and accompanied by greater inter-fish distance than WT zebrafish. The aggression test showed a reduced aggressive behavior in Neu1-KO zebrafish than in WT zebrafish. In the mirror and 3-chambers test, Neu1-KO zebrafish showed more interest toward the opponent in the mirror and multiple unfamiliar zebrafish, respectively, than WT zebrafish. Furthermore, Neu1-KO zebrafish also showed increased interaction with different fish species, whereas WT zebrafish avoided them. In the black–white preference test, Neu1-KO zebrafish showed an abnormal preference for the white region, whereas WT zebrafish preferred the black region. Neu1-KO zebrafish were characterized by a downregulation of the anxiety-related genes of the hypothalamic–pituitary–adrenal axis and upregulation of *lamp1a*, an activator of lysosomal exocytosis, with their brains accumulating several sphingoglycolipids. This study revealed that Neu1 deficiency caused abnormal emotional behavior in zebrafish, possibly due to neuronal dysfunction induced by lysosomal exocytosis.

## Introduction

Lysosomal disorders are identified as incurable diseases, affecting a very small number of patients, namely 1 in 5000^1^. Approximately 70 types of lysosomal diseases have been reported and shown to be genetically induced by gene mutations in lysosomal-related proteins, mostly catabolic enzymes and lysosome membrane proteins^1^. Dysfunction of lysosomal enzymes in patients is known to result in the accumulation of non-degraded substrates, thus inducing a reduction in lysosomal functions.

Sialidosis (Online Mendelian Inheritance in Man (OMIM) #256550) is a lysosomal disease with low incidence (1:250,000 to 1:2,000,000 live births), classified into type I (milder symptoms) and type II (severe symptoms), which can be subdivided into 3 forms: congenital, infantile, and juvenile^2,3^. Symptoms of patients with sialidosis include myoclonus, spinal deformities, cherry red spots, inner ear hearing loss, hepatosplenomegaly, and visual failure^4,5^. In addition, patients with sialidosis have been reported to show psychiatric abnormalities, such as mental decline, low IQ, and intellectual disability^2,6^.

Sialidosis is caused by a genomic DNA mutation in neuraminidase 1 (*NEU1*) (chromosome location: 6p 21.3). The NEU1 protein is a sialidase (EC 3.2.1.18), a representative of the lysosomal glycosidases that cleaves sialic acids from the nonreducing ends of glycoconjugates, mainly from oligosaccharides and glycoproteins^7^. In lysosomes, desialylation of oligosaccharides by NEU1 is known to be crucial in regulating the initial step in the degradation of sugar chains. A part of NEU1 localizes to the plasma membrane, where NEU1 regulates cell attachment, insulin signaling, and elastin binding^8–10^. The enzymatic activity of NEU1 is activated/stabilized by cathepsin A. Cathepsin A dysfunctions due to mutations in *CTSA* (cathepsin A gene) causes galactosialidosis (OMIM # 256540), which is characterized by similar symptoms to those of sialidosis. Several DNA mutations in the *NEU1* gene in patients with sialidosis have been reported^2,11^, with these mutations inducing alterations in the activity of sialidase, lysosomal localization, complex formation, structure, and half-life of NEU1 polypeptides^12–14^, thus leading to a significant impact on the symptoms of the disease.

So far, both SM/J and Neu1-KO mice have been used for sialidosis studies. SM/J mice are known to possess a single substitution mutation (L209I) in the NEU1 protein, leading to a decrease in the activity of lysosomal sialidase, smaller body size, and possible alterations of immune responses^15^. Likewise, Neu1-KO mice exhibit a smaller body, the curvature of the cervical spine, induction of vacuolation in the kidneys, and an enlarged spleen^16^. Using rodent models, the molecular mechanism of the incidence of sialidosis symptoms has been partially identified. For instance, excessive exocytosis of serine proteases in the bone niche has been reported to lead to loss of bone marrow retention^17^. The activated components of the transforming growth factor β (TGF-β) and wingless-related integration site (WNT) signaling pathways in the exosome are involved in the abnormal fibrotic process in muscle connective tissue fibroblasts^18^.

While the mechanism of the dysfunction of the NEU1 deficiency-induced bone and muscle formation has been sufficiently studied, little is known about the involvement of NEU1 in emotional regulation, unlike other lysosomal diseases. For instance, patients with Fabry disease are known to show mood disorders, including anxiety and depression, whereas patients with Niemann-Pick disease type C show psychiatric symptoms, including hallucinations, delusions, cognitive decline, dementia, depression, bipolar disorder, as well as disruptive and aggressive behavior^19^. In addition, patients with α-mannosidosis, mucopolysaccharidosis, and Tay–Sachs disease have also been reported to exhibit mood disorders^20^. As lysosomal diseases have been shown to share many common symptoms and sialic acid is known to be present in high levels in central nerves^21^, we hypothesized that a NEU1 deficiency might affect emotional and social interactions.

Here, we focused on teleost to examine the involvement of NEU1 in emotional behavior. Fish are excellent experimental animals for the analysis of social interaction, including group formation, aggressiveness, social preference, and boldness. Most emotional related-genes are known to be conserved across vertebrates, and several methodologies of behavioral analysis have been established in fish^22,23^. In addition, recent studies have revealed that *neu1* genes are conserved in fish species, with their enzymatic properties^24–27^. Fish Neu1 is known to be localized at the lysosome and its enzymatic properties, such as optimal pH and substrate specificity, have been reported to be shared among fish species. Recently, we established a Neu1 knockout zebrafish (Neu1-KO) strain using the CRISPR/Cas9 genome editing method^26^. The Neu1-KO zebrafish was shown to possess 5 nucleotide deletions in the first exon, which caused a frameshift, resulting in the interruption of the translation of the Neu1 polypeptide. The Neu1-KO zebrafish was characterized by a smaller body size with the bending spine similar to patients with sialidosis and Neu1-KO mice. This study aimed to reveal the effects of a Neu1-deficiency on the behavior of Neu1-KO zebrafish.

## Results

### Neu1-KO zebrafish exhibited lower social interaction and aggression

**Figure 1:**
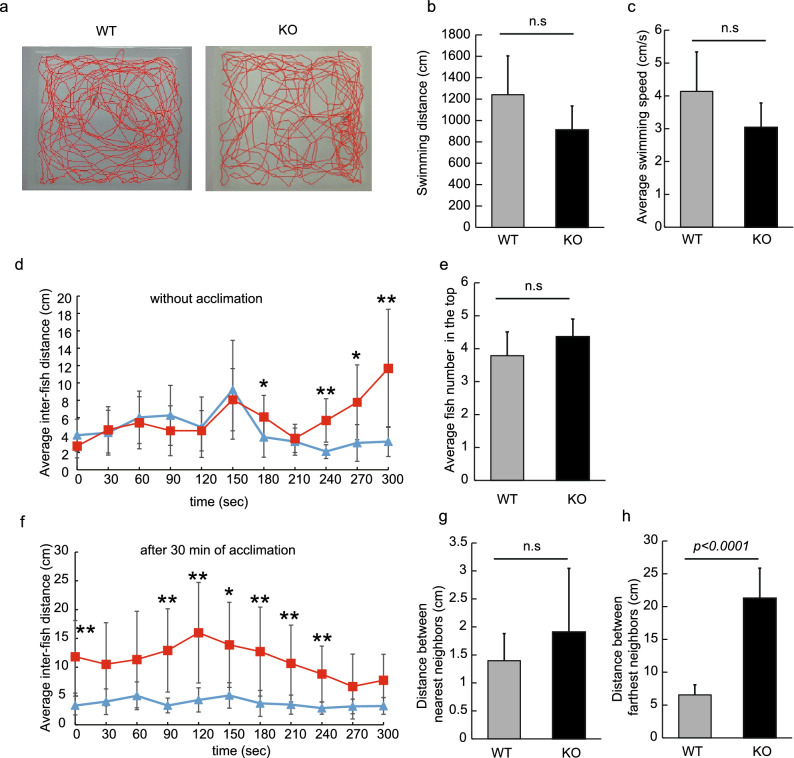
Analysis of shoaling in Neu1-KO and WT zebrafish. (**a**–**c**) Analysis of the behavior of Neu1-KO zebrafish (6 months) in the normal tank for 5 min. (**a**) Tracking. (**b**) Swimming distance. (**c**) Average swimming speed. WT, wild-type and KO, Neu1-KO zebrafish. n = 7 for each group. (**d**–**h**) Shoaling of Neu1-KO zebrafish. Five fish were set in the experimental tank, and their average inter-fish distance was immediately estimated (**d**, without acclimation) . After 30 min acclimation in the test tank, inter-fish distances were evaluated again (**f**, with acclimation). Closed blue triangles and red squares with single line indicate WT and KO, respectively. (**e**) Average fish number in the top area for 5 min. (**g**) Average distance between nearest and (**h**) farthest neighbors analyzed for 5 min in WT and Neu1-KO zebrafish during the shoaling test without acclimation. Results are shown as means ± standard deviation. All values were compared using Mann–Whitney U test. *p < 0.05, **p < 0.01. n.s., not significant.

A previous study reported that older Neu1-KO zebrafish (> 11 months old) exhibited spinal bending, inducing a suppression in their swimming ability^26^. Thus, this study employed younger Neu1-KO zebrafish that had not developed any such symptoms and were considered suitable for behavioral analysis. To confirm the swimming ability of young adult Neu1-KO zebrafish, 6-months fish were transferred to the normal tank and their swimming behavior was observed after 5 min of acclimation. We found that Neu1-KO zebrafish did not exhibit any abnormal behavior, such as freezing and limited swimming along the tank wall, and their swimming distance and average swimming speed did not differ from those of WT (Fig. 1a–c).

We conducted the shoaling test with Neu1-KO zebrafish using the novel tank apparatus. Shoaling is thought to provide each fish with multiple benefits, including access to mates, efficient foraging, and defense against predators^28^. First, 5 zebrafish were transferred to a novel tank, and then the estimation of their inter-fish distance was performed every 30 s immediately after the start of the test without acclimation. The inter-fish distance is known to represent the shoaling formation. We observed that both WT and Neu1-KO zebrafish formed shoaling after their transfer to the tank, probably due to anxiety. However, after 240 s, the inter-fish distance in Neu1-KO zebrafish was demonstrated to be significantly increased compared with that of WT (*p* < 0.01, Fig. 1d). Next, to remove the effect of anxiety induced by the novel tank, fish were acclimated for 30 min and then used for behavioral assays. In general, the number of fish in the top reflects the place preference of the shoal, which is likely to be near the bottom for more anxious fish. In Neu1-KO zebrafish, the place preference of shoal did not differ from WT (Fig. 1e). We found that under acclimation conditions, the increased fish intervals were sustained in Neu1-KO zebrafish compared with those of WT zebrafish (*p* < 0.01, Fig. 1f). The distance between the farthest neighbors was significantly longer in the Neu1-KO zebrafish relative to that in WT (3.5-fold increase, *p* < 0.0001, Fig. 1h), whereas the distance between the nearest neighbors was not (Fig. 1g). These results indicated low shoaling in Neu1-KO zebrafish.

**Figure 2:**
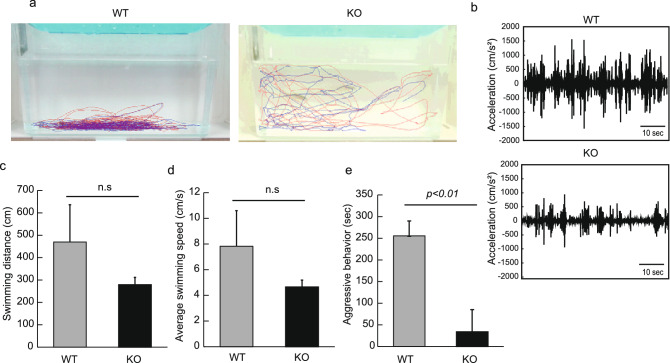
Analysis of aggressive behavior in Neu1-KO and WT zebrafish. Two unfamiliar male zebrafish were set in the transparent aquarium and their aggressive behavior was observed for 10 min. (**a**) Tracking of 2 males in WT and Neu1-KO. Red and blue lines indicate the swimming tracking of 2 males. (**b**) Swimming acceleration. (**c**) Swimming distance. (**d**) Swimming speed. (**e**) Aggressive behavior. n = 6 for each group. Results are shown as means ± standard deviation. All values were compared using Mann–Whitney U test. n.s., not significant.

Next, we assessed the aggressiveness of Neu1-KO zebrafish. We noted that WT zebrafish started continuous chasing accompanied by the rapid acceleration of swimming (Fig. 2a,b). In contrast, Neu1-KO zebrafish seemed indifferent to each other and swam independently exhibiting slight chasing behavior. According to the induction of rapid acceleration of swimming, we found that WT zebrafish tended to have longer swimming distance and faster swimming speed than Neu1-KO zebrafish. (Fig. 2c,d). During a 10 min observation, WT zebrafish exhibited a significantly longer aggressive behavior (total average: 255.5 s) than Neu1-KO zebrafish (average 34.0 s, *p* < 0.01, Fig. 2e). These results suggested a low aggressiveness in Neu1-KO zebrafish.

### Neu1 knockout zebrafish showed abnormal social preferences

**Figure 3:**
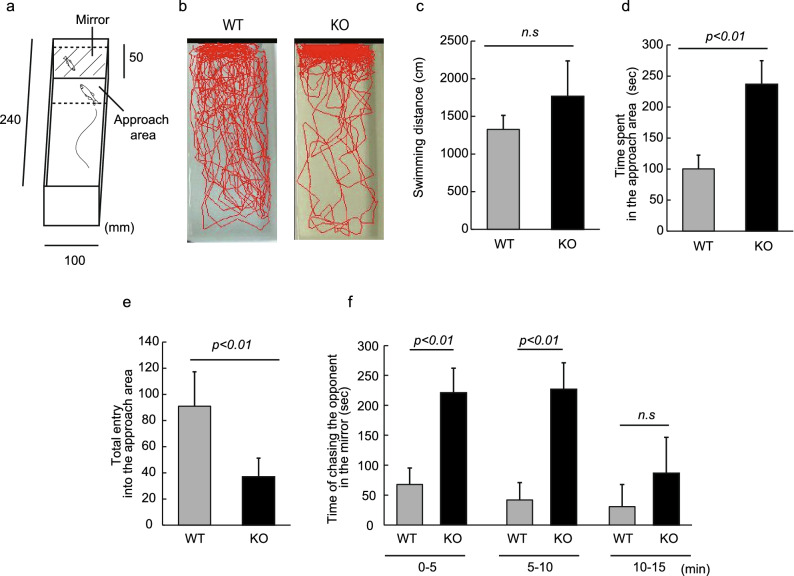
Analysis of Neu1-KO and WT zebrafish behavior in a mirror test. (**a**) Apparatus used in the mirror test. (**b**) Tracking of WT and Neu1-KO zebrafish. (**c**) Swimming distance. (**d**) Time spent in the approach area. (**e**) Frequency of entry into the approach area. (**f**) Time of chasing the opponent in the mirror. n = 10 for each group. Results are shown as means ± standard deviations. All values were compared using Mann–Whitney U test. n.s., not significant.

The mirror test is generally used for studying the sociality, aggressiveness, and boldness of fish (Fig. 3a). In the mirror test, test fish are known to recognize the opponent in the mirror as an unfamiliar zebrafish. Here, we employed the mirror test on Neu1-KO zebrafish to evaluate their emotional responses. As a result, we found that the swimming behaviors of Neu1-KO zebrafish were drastically different from those of WT zebrafish. Both WT and Neu1-KO zebrafish showed an interaction with the mirror (towards the opponent in the mirror), but Neu1-KO zebrafish were noted to approach the mirror more than WT zebrafish (Fig. 3b), despite exhibiting a similar total swimming distance to that of WT (Fig. 3c). This result indicated that the increased interaction observed in Neu1-KO zebrafish might not be due to hyperactivity or impulsive behavior. We also observed that Neu1-KO zebrafish exhibited a significant increase in the total time in the approach area (2.4-times longer than WT, *p *< 0.01, Fig. 3d). However, the total entry into the approach area in Neu1-KO zebrafish was shown to be less than that in WT (*p *< 0.01, Fig. 3e), indicating that Neu1-KO zebrafish stayed for a longer period in the approach area in a single invasion compared with WT zebrafish. After approaching the mirror, the zebrafish showed a chasing behavior towards the opponent (itself in the mirror). We found that the chasing time in Neu1-KO zebrafish was significantly longer than that in WT zebrafish during the first 10 min after the beginning of analysis (*p* < 0.01), whereas dropped to the WT level after 10 min (Fig. 3f). Taken together with the results of Figures 1 and 2, Neu1-KO zebrafish exhibited increased interest toward the opponent in the mirror, but not due to shoaling and aggressiveness.

**Figure 4:**
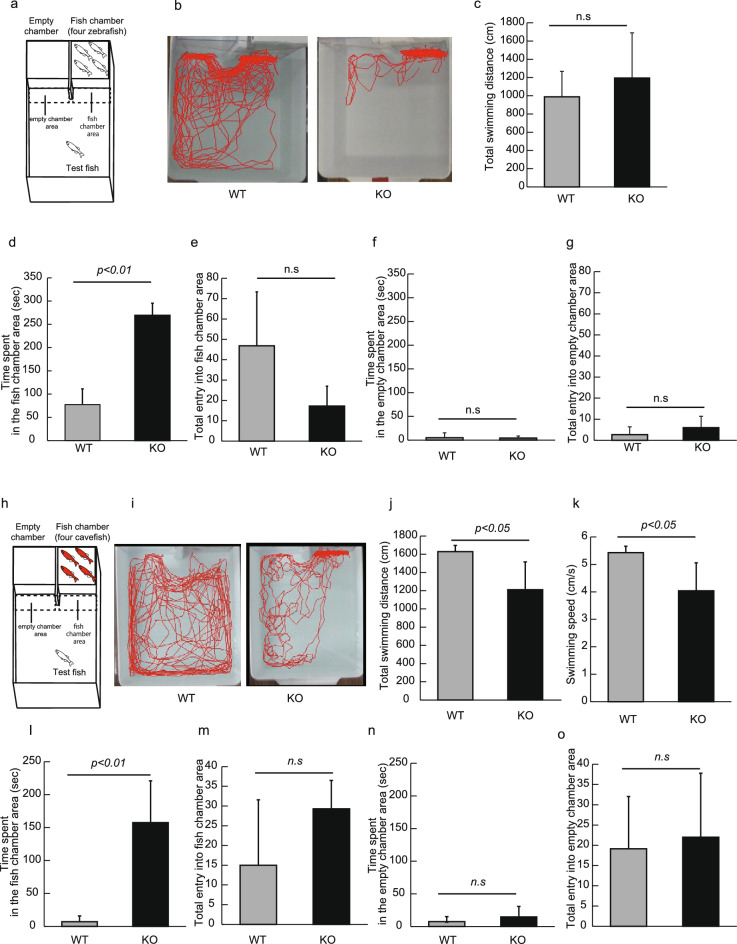
Analysis of social preference in Neu1-KO and WT zebrafish using the 3-chambers test. (**a**–**g**) Analysis of the 3-chambers test with 4 unfamiliar zebrafish in Neu1-KO and WT zebrafish. (**a**) Apparatus of the 3-chambers test using 4 zebrafish. (**b**) Tracking of WT and Neu1-KO zebrafish. (**c**) Total swimming distance. (**d**) Time spent in the fish chamber area. (**e**) Total entry into the fish chamber area. (**f**) Time spent in the empty chamber area. (**g**) Total entry into the empty chamber area. n = 8 for each group. (**h**–**o**) Analysis of the 3-chambers test with 4 cavefish in Neu1-KO and WT zebrafish. (**h**) Apparatus of the 3-chambers test with 4 cavefish. (**i**) Tracking of WT and Neu1-KO zebrafish. (**j**) Total swimming distance. (**k**) Swimming speed. (**l**) Time spent in the fish chamber area. (**m**) Total entry into fish chamber area. (**n**) Time spent in the empty chamber area. (**o**) Total entry into empty chamber area. n = 10 for each group. Results are shown as means ± standard deviation. All values were compared using Mann–Whitney U test. n.s., not significant.

To better understand the interaction of Neu1-KO zebrafish with unfamiliar zebrafish, we conducted a 3-chambers test using 2 chambers: an empty chamber and a chamber containing 4 unfamiliar zebrafish that had been kept in a different aquarium from test fish (Fig. 4a). During the 5 min trial, we found that WT zebrafish swam throughout the tank, showing interest in both chambers, and especially in the zebrafish tank (Fig. 4b). We observed that WT zebrafish swam along the front wall of the chambers after their entry into the fish chamber area. In contrast, we found that the swimming trajectory in Neu1-KO zebrafish showed limited locomotion in the fish chamber area accompanied by a remarkable swimming along the chamber wall (Fig. 4b). Whereas both WT and Neu1-KO zebrafish covered a similar swimming distance (Fig. 4c), the time spent in the chamber area was 4.0-fold longer in Neu1-KO zebrafish than in WT (*p* < 0.01, Fig. 4d). However, we noted that the frequency of entry into the fish chamber area did not differ between WT and Neu1-KO zebrafish (Fig. 4e). Unlike the differences noted regarding the fish chamber, both WT and Neu1-KO zebrafish showed similar responses toward the empty chamber (Fig. 4f,g).

Next, we estimated the social preference for different fish species using cavefish (Mexican tetra, Fig. 4h). Cavefish is an unfamiliar fish for zebrafish, with a red body color distinctive from zebrafish. In addition, as cavefish are blinded due to degeneration of their eyes, the effects of the behavior of zebrafish on cavefish could be ignored. We observed that WT zebrafish showed vigilance and anxiety toward cavefish and swam far from the chamber, whereas sometimes invaded the fish chamber area probably due to interest/exploration, which was however followed by rapid escape from the chamber area (Fig. 4i). In contrast, Neu1-KO zebrafish showed a vigilant behavior only at the beginning of the trial and often invaded the fish chamber area (Fig. 4i). Due to their vigilant action, we recorded longer swimming distance and higher average velocity in WT compared with Neu1-KO zebrafish (*p* < 0.05, Fig. 4j,k). Based on their increased interest toward cavefish, the time spent in the fish chamber was shown to be drastically increased in Neu1-KO zebrafish (15-folds increase, *p* < 0.01, Fig. 4l), although the total entry into the fish chamber area was not different from that of WT (Fig. 4m). Both WT and Neu1-KO zebrafish exhibited similar responses to the empty chamber (Fig. 4n,o). Taken together, Neu1-KO zebrafish exhibited an abnormal social preference toward unfamiliar fish, which was seemingly attributed to their excessive high boldness or abnormal recognition.

**Figure 5:**
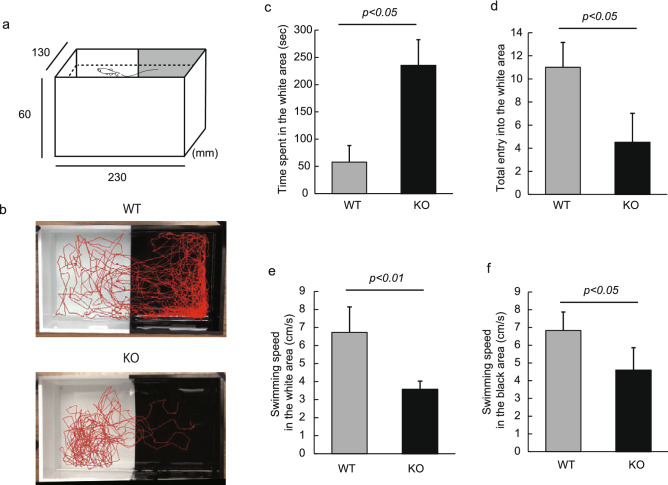
Analysis of the black–white preference test in Neu1-KO and WT zebrafish. (**a**) Apparatus in the black–white preference test. (**b**) Tracking of WT (upper panel) and Neu1-KO zebrafish behavior (lower panel). (**c**) Time spent in the white area. (**d**) Total entry into the white area. (**e**,**f**) Swimming speed in white (**e**) and black area (**f**). n = 5 for each group. Results are shown as means ± standard deviation. All values were compared using Mann–Whitney U test.

For further analysis of the boldness/recognition behavior of Neu1-KO zebrafish, we carried out the black–white preference test (Fig. 5a). In general, adult zebrafish are known to instinctively prefer black to white areas because fish have a low risk of being detected by predators in a black background^23^. As expected, we noted that WT zebrafish stayed in the black area longer than in the white area during the 5 min analysis (Fig. 5b). In contrast, Neu1-KO zebrafish were found to obviously prefer the white to the black areas (Fig. 5b). The time spent by Neu1-KO zebrafish in the white area was significantly increased compared with that of WT (4.6-fold increase, *p* < 0.05, Fig. 5c), with the total entry of Neu1-KO zebrafish into the white area being less than that observed in WT (2.8-fold decrease, *p* < 0.05, Fig. 5d), indicating that Neu1-KO zebrafish stayed on the white side for a longer time per invasion. In addition, zebrafish are known to feel anxious toward the white color^23^. As shown in Fig. 5b, WT zebrafish showed anxiety accompanied by swimming along the wall and erratic movements/zig-zagging, which was not observed in Neu1-KO zebrafish. We further observed that the anxiety of WT fish induced a higher swimming speed compared with that exhibited by Neu1-KO zebrafish in both the black and white areas. (Fig. 5e,f).

**Figure 6:**
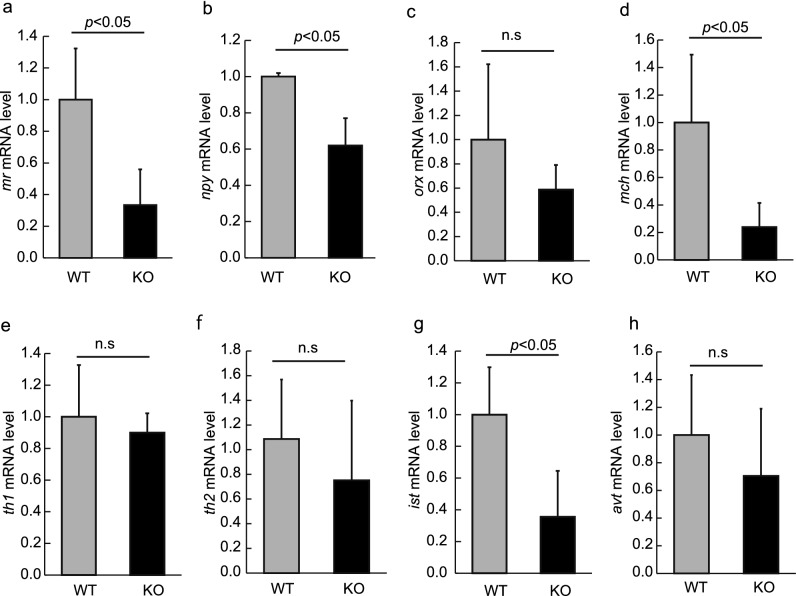
Changes in gene expression in Neu1-KO zebrafish. mRNA levels of *mr* (**a**), *npy* (**b**), *orx* (**c**), *mch* (**d**), *th1* (**e**), *th2* (**f**), *ist* (**g**), and *avt* (**h**) in WT and Neu1-KO zebrafish. Each level of gene expression in Neu1-KO zebrafish is relative to the value in WT fish. n = 5 for each group. Results are shown as means ± standard deviation. All values were compared using Mann–Whitney U test. n.s., not significant.

To examine the alteration of emotions in Neu1-KO zebrafish, we estimated the mRNA levels of anxiety-related genes in WT and Neu1-KO zebrafish brains. The mineralocorticoid receptor (Mr), which is also conserved in zebrafish^23^, is known to regulate the activity of the hypothalamic–pituitary–adrenal (HPA) axis during the stress response^29^. We found that the *mr* mRNA level was significantly downregulated in Neu1-KO zebrafish compared with that in WT (2.6-fold decrease, *p* < 0.05, Fig. 6a). Neuropeptide Y is a downstream molecule of the Mr signaling through negative feedback^30^ and is known to be expressed at the locus coeruleus (LC) in zebrafish^23^. We noted that the mRNA level of *npy* was also significantly reduced in Neu1-KO zebrafish (*p* < 0.05, Fig. 6b). While both orexin (Orx) and melanin-concentrating hormone (Mch) are known as anxiogenic factors through the stimulation of the HPA axis, only the Mch mRNA levels were shown to be downregulated in Neu1-KO zebrafish brains compared with WT (*p* < 0.05, Fig. 6c,d). The sympathetic–adrenal–medullary (SAM) axis has also been reported to regulate anxiety behaviors through the elevation of the levels of catecholamine in vertebrates. The present study evaluated the gene expression of tyrosine hydroxylase (*th*), which converts tyrosine to dihydroxyphenylalanine, a precursor of dopamine and noradrenaline, for which zebrafish are known to possess 2 isoforms (Th1 and Th2)^31^. As a result, the levels of *th1* and *th2* in Neu1-KO zebrafish were not altered compared with WT (Fig. 6e,f). Taken together, Neu1-KO zebrafish showed an excess increased interest/exploratory activity, possibly due to decreased anxiety accompanied by the downregulation of the HPA axis signaling. Isotocin (Ist, teleost homologues of oxytocin) is a factor known to positively regulates societal and negative aggressiveness^32^, whereas vasotocin (Avt, teleost homologues of vasopressin) accelerates aggressiveness in zebrafish^33^. We found that the expression of *ist* in the brain of Neu1-KO zebrafish was significantly higher than that in WT (*p* < 0.05), whereas the levels of *avt* mRNA did not differ between Neu1-KO and WT zebrafish (Fig. 6g,h), confirming that Neu1 deficiency caused low shoaling and aggressiveness in zebrafish.

### Neu1-KO zebrafish exhibited an accumulation of sphingoglycolipids in the brain

**Figure 7:**
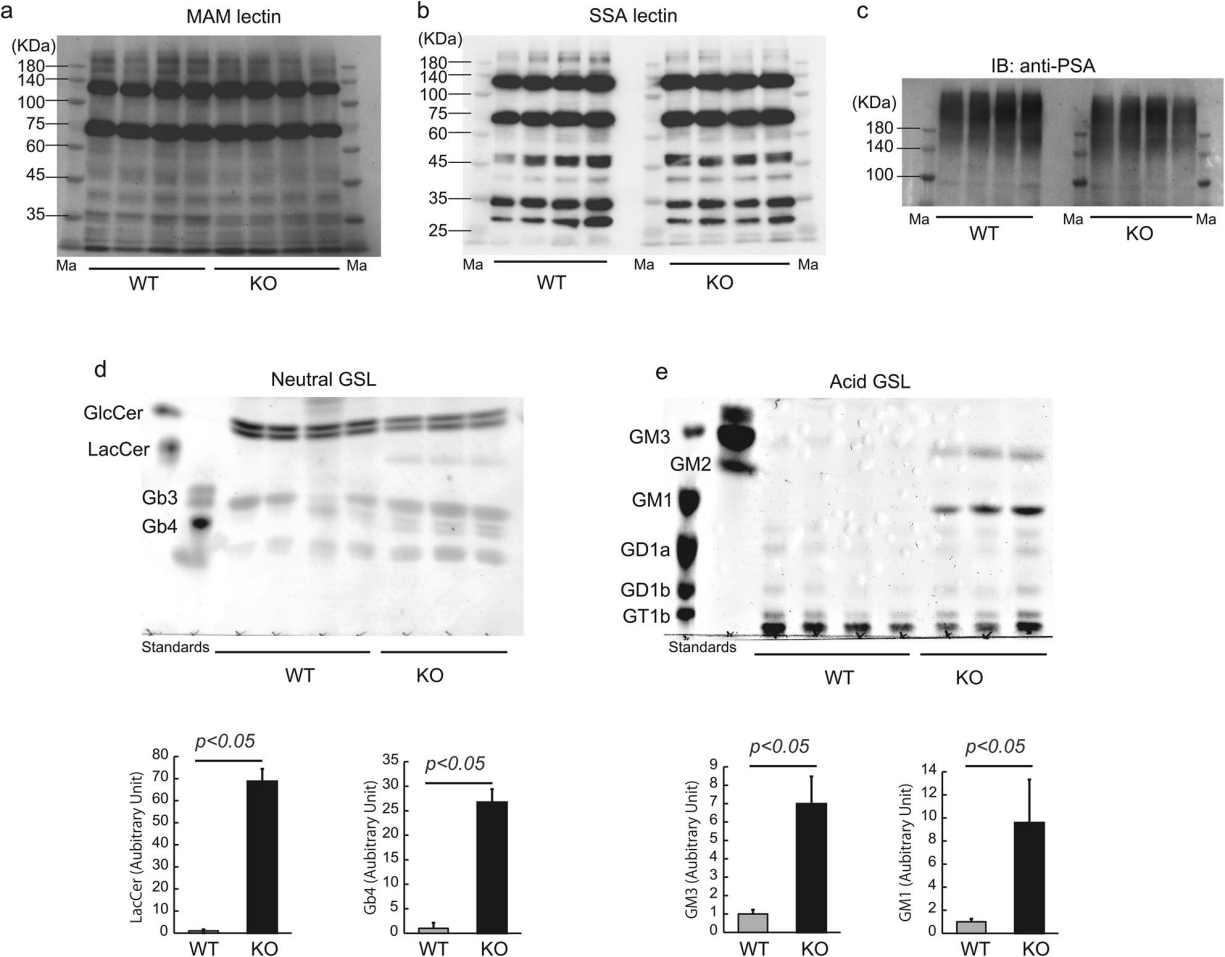
Alteration of glycoconjugates patterns in Neu1-KO zebrafish. (**a**–**c**) Lysates of zebrafish brains were applied for lectin and western blotting. (**a**) MAM lectin. (**b**) SSA lectin. (**c**) Anti-PSA immunoblotting. Ma, Molecular marker. (**d**,**e**) GSLs were extracted from zebrafish brains and fractionated into neutral and acid fractions. Two fractions were applied for thin layer chromatography and GSLs were detected using orcinol-H_2_SO_4_. Neutral (**d**) and acid fraction (**e**) of GSLs. Results are shown as means ± standard deviation. All values were compared using Mann–Whitney U test.

We assessed the alterations of sialoglycoconjugates in Neu1-KO zebrafish brain by lectin blotting using MAM (Sia α2-3 linkage specific) and SSA lectin (Sia α2-6 linkage specific). Although the recombinant zebrafish Neu1 has been reported to recognize both Sia α2-3 and α2-6 linkages in oligosaccharides and glycoproteins^26^, we could not detect a clear difference in the glycosylation pattern between Neu1-KO and WT zebrafish brains in our MAM and SSA lectin blot analysis (Fig. 7a,b). In mammals, Neu1 is known to desialyze polysialic acids (PSA) accompanied by a polysialic acid turnover in the regulation of neurotrophin^34^ and lamination of newly generated hippocampal granule cells^35^. However, we observed that the PSA pattern in Neu1-KO zebrafish brains did not differ from that of WT (Fig. 7c and Supplementary Fig. 1). Interestingly, the glycosphingolipid (GSL) pattern in the Neu1-KO zebrafish brain was shown to drastically differ from that in the WT brain, despite gangliosides not being substrates for zebrafish Neu1^26^. TLC analysis of the neutral lipid fraction showed an accumulation of lactosylceramide (LacCer) and globoside Gb4 in Neu1-KO zebrafish, whereas these two GSLs were almost undetectable in WT (*p* < 0.05, Fig. 7d). We further noted that the GSL pattern in the acid fraction exhibited an increase in GM3 and GM1 in the Neu1-KO zebrafish brain (*p* < 0.05), but not in the WT, whereas the levels of GD1b and GT1b were almost the same between Neu1-KO and WT zebrafish brains (Fig. 7e).

**Figure 8:**
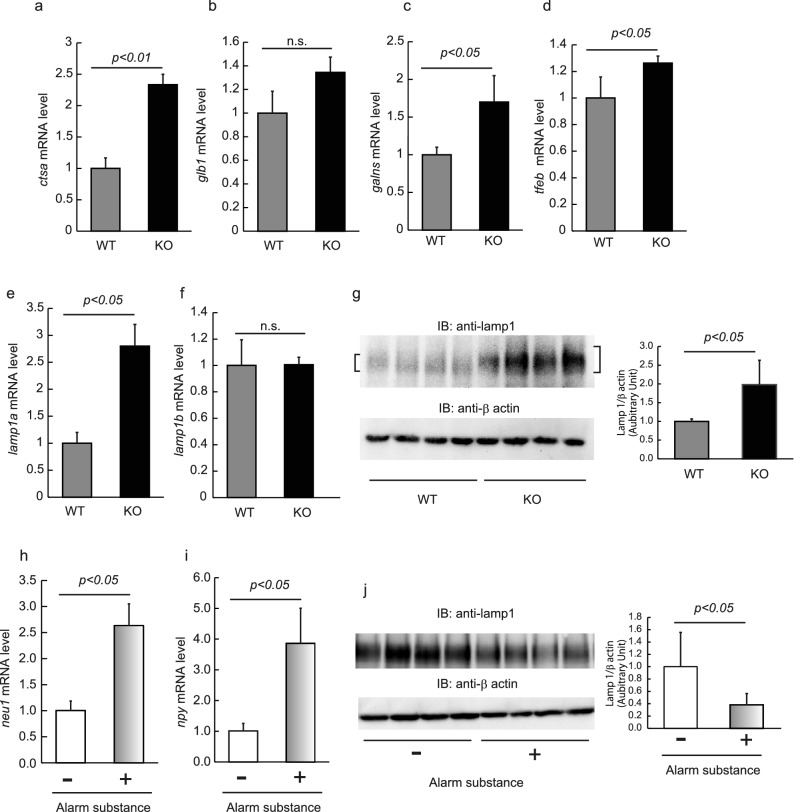
Changes in the expression of lysosomal genes in Neu1-KO zebrafish. mRNA levels of *ctsa* (**a**), *glb1* (**b**), *galns* (**c**), *tfeb* (**d**), *lamp1a* (**e**), and *lamp1b* (**f**) in WT and Neu1-KO zebrafish. Each level of gene expression in Neu1-KO zebrafish is relative to the value in WT fish. n = 7 for each group. (**g**) Lysates of zebrafish brains were applied for western blotting using anti-lamp1 and anti-β actin antibodies. Quantitative analyses of the intensities of Lamp1 and β-actin bands were carried out and results are presented as Lamp1/β-actin. (**h**–**j**) WT zebrafish were exposed to alarm substances and then the *neu1* (**h**) and *npy* mRNA levels (**i**) were estimated by real-time PCR. Lysates of zebrafish brains were applied for western blotting using anti-lamp1 and anti-β actin antibodies (**j**). Quantitative analyses of the intensities of Lamp1 and β-actin bands were carried out and results are presented as Lamp1/β-actin. n = 6 for each group. Results are shown as means ± standard deviation. All values were compared using Mann–Whitney U test. n.s., not significant.

Furthermore, we focused on the lysosomal conditions in Neu1-KO zebrafish. Neu1 is known to form a multienzyme complex with cathepsin A, β-galactosidase, and *N*-acetylgalactosamine-6-sulfatase^36^. Due to the deficiency of Neu1, the *ctsa* and *galns* mRNA levels were shown to be significantly increased in Neu1-KO zebrafish brains compared with WT (*p* < 0.01 for *ctsa*, and *p* < 0.05 for *galns*); however, that was not the case for *glb1* (Fig. 8a–c). Transcription factor EB (Tfeb) is known as a master regulator of mammalian lysosomal genes, including *Neu1* and *Ctsa*^37^. As expected, the Neu1-KO zebrafish brain showed higher *tfeb* mRNA levels compared with the WT brain (*p* < 0.05, Fig. 8d) accompanied by an upregulation of *lamp1a* (*p* < 0.05), but not *lamp1b* (Fig. 8e,f), showing the same pattern as Neu1-KO zebrafish muscle^26^. In mammals, TFEB is known to activate the translocalization of lysosomal enzymes to the plasma membrane^38^, with NEU1 being a negative regulator of lysosomal exocytosis through the desialylation of LAMP1^17^. Immunoblot analysis revealed that the level of the Lamp1 protein was upregulated in Neu1-KO zebrafish brain compared with WT, coinciding with the *lamp1a* mRNA level (*p* < 0.05, Fig. 8g and Supplementary Fig. 2). Moreover, the observed molecular weight of Lamp1 in the Neu1-KO brain was shown to be higher than that in the WT brain, possibly due to glycosylation similar to that observed in Neu1-KO mice (Fig. 8g). These results suggested the possible induction of lysosome exocytosis and neuronal dysfunction in Neu1-KO zebrafish. To confirm the involvement of lysosomal exocytosis in zebrafish behavior, we exposed WT zebrafish to alarm substances, which is known to evoke an anxiety behavior and suppress bold behaviors, and evaluated the levels of the Lamp1 protein in the brain. We accordingly found that zebrafish exposed to alarm substances showed a significant upregulation of *neu1* in the brain compared with non-exposed fish (*p* < 0.05, Fig. 8h). Moreover, the *npy* mRNA level in the treated zebrafish brain confirmed the induction of anxiety via the HPA axis (*p* < 0.05, Fig. 8i). In addition, we noted that the level of the Lamp1 protein in alarm substance-exposed zebrafish was significantly decreased compared with that in non-exposed zebrafish (42% decrease relative to non-exposed, *p* < 0.05, Fig. 8j and Supplementary Fig. 2). These results suggested that upregulation of the *neu1* gene during zebrafish anxiety suppressed lysosomal exocytosis, leading to a reduction in exploratory/boldness behavior.

## Discussion

The deficiency in NEU1 sialidase is responsible for human sialidosis^7^. Although the mechanisms of muscle and bone dysfunctions in sialidosis have been clarified, the role of NEU1 in the regulation of emotional activity has not been explored to this day. We accordingly found that Neu1-KO zebrafish exhibited excess boldness/exploratory behavior in various tests, accompanied by a decrease in the expression of anxiety-related genes. In addition, we observed that lysosomal exocytosis was enhanced in Neu1-KO zebrafish.

First, the present study revealed the low shoaling of Neu1-KO zebrafish. The low shoaling behavior in Neu1-KO fish indicated an anxiety reduction. Moreover, Neu1-KO zebrafish exhibited a drastic alteration in the expression of emotional genes, especially in the HPA axis. In general, a decreased HPA axis has been considered as a low anxiety condition in zebrafish^39^. In addition, we noted that Neu1-KO zebrafish showed high interaction with the "unfamiliar" opponent in the mirror. As Neu1-KO zebrafish showed low aggression toward the unfamiliar zebrafish, the induction of a high interaction in the mirror test in Neu1-KO zebrafish was assumed to be caused by their increased interest toward the unfamiliar zebrafish. Neu1-KO zebrafish exhibited high interaction not only toward multiple unfamiliar zebrafish but also toward an unfamiliar fish species, cavefish. Interestingly, zebrafish are known to show strong avoidance behavior toward unfamiliar fish species^40^. Taken together, Neu1-KO zebrafish were determined to be bold, but not impulsive, because we did not observe any hyperactivity (enhancement of locomotion) in Neu1-KO zebrafish, as in the case of patients with sialidosis^41^. Chronic stress and dopamine/noradrenalin neurons are also known to be involved in boldness^23^; however, Neu1-KO zebrafish did not exhibit any alterations in the expression of tyrosine hydroxylase genes. Considering the instincts characterizing all living animals, excess boldness/exploratory behavior must be avoided to protect life. As such, anxiety-related genes play a role in interrupting the excessive activity under situations associated with potential danger. In this study, we found that Neu1-KO zebrafish showed increased interactions with unfamiliar zebrafish, different fish species, and white areas, without exhibiting aggression or anxiety, although these were factors that should have been handled with caution. Thus, we concluded that Neu1-KO zebrafish show abnormal behavior. Furthermore, induction of anxiety evoked the upregulation of *neu1* in the zebrafish brain accompanied by reduced levels of Lamp1, suggesting that the significance of enhanced Neu1 and reduced lysosomal exocytosis might be to inhibit the boldness/exploratory activity under situations that require caution.

The level of Lamp1 was shown to be upregulated in the Neu1-KO zebrafish brain, and its molecular weight was found to be higher than that of WT, possibly due to glycosylation. Similarly, Neu1-KO mice have been reported to show excessive lysosomal exocytosis in neural cells accompanied by increased levels of Lamp1^17^. Lysosomal exocytosis observed in NEU1 deficiency has been suggested to be deeply involved in the symptoms of patients with sialidosis and Neu1-KO mice, in contrast to other lysosomal diseases accompanied by lysosome expansion. Patients with sialidosis have been reported to show muscle hypotonia, similar to Neu1-KO mice with skeletal muscle atrophy accompanied by an expansion of the epimysial and perimysial spaces^42^. Neu1-deficiency has been shown to cause the production of excessive lysosomes that contain activated TGF-β and WNT/β-catenin signaling molecules, leading to the conversion of fibroblasts into myofibroblasts^18^. A previous study reported that upregulation of *lamp1a* occurred also in the muscles of Neu1-KO zebrafish^26^, suggesting the induction of exocytosis in the whole Neu1-KO zebrafish body. However, the involvement of lysosomal exocytosis in emotional dysregulation in Neu1-KO zebrafish remains unclear. Recently, the long-term structural plasticity of dendritic spines was reported to be regulated in the hippocampus by lysosomal exocytosis, accompanied by increased activity of cathepsin B^43^. One of the possible reasons behind the abnormal behavior of Neu1-KO zebrafish might be the dysfunction of short-term memory. More specifically, the behavior of animals expressing low exploratory activity in a novel tank exploration test and increased swimming speed in the mirror test, and not showing any fear response to unfamiliar fish species are thought to be related to the dysfunction of short-term memory^44^. Furthermore, the involvement of sialidase in hippocampal neuronal cells has also been explored. For instance, the activity of sialidase was shown to be increased in response to neuronal excitation in the rat hippocampus^45^. Moreover, increased release of sialic acid has been detected during in vivo microdialysis during fear conditioning. Furthermore, Neu1-KO mice were reported to accumulate amyloid-beta (Aβ) in the CA3 region of the hippocampus, induced by enhanced lysosomal exocytosis^46^. The accumulation of Aβ has been suggested to be involved in the development of Alzheimer’s disease, with impaired cognition being one of the major symptoms of the disease. In addition, the present study showed the abnormal accumulation of GM1 ganglioside in the brain of Neu1-KO zebrafish. In particular, GM1 is known to promote the accumulation of Aβ^47^. These findings suggested that Neu1-KO zebrafish might exhibit Alzheimer’s disease-like symptoms, inducing this abnormal behavior. Further studies are needed to clarify whether dysfunctions in short-term memory and memory maintenance are involved in the behavior of Neu1-KO zebrafish.

We did not observe any significant accumulation of sialoglycoproteins in Neu1-KO zebrafish brains, unlike Neu1-KO zebrafish larvae^26^. It should be mentioned that PSA is known to regulate neurogenesis, memory, and abnormal PSA profiles have been reported to induce autism spectrum and bipolar disorders^21^. We found that the levels of PSA in Neu1-KO zebrafish were not different from those in WT, although mammalian Neu1 is known to desialyze PSA. In contrast, Neu1-KO zebrafish were shown to accumulate some GSLs in the brain, such as GM1, GM3, LacCer, and Gb4. However, these accumulations in Neu1-KO zebrafish could not be caused by the deficiency of the activity of the Neu1 enzyme because gangliosides are not substrates for zebrafish Neu1^26^. A similar accumulation of gangliosides has been found in the brains of mice in several lysosomal diseases^48^. For example, GM1, GM2, GM3, and GD3 were reported to be accumulated in Gaucher disease. Despite this finding, the relationship between gangliosides and abnormal animal behavior has not been fully clarified. Recently, the involvement of Neu3 and Neu4 in short-term memory was assessed using Neu3^−/−^/Neu4^−/−^ double knockout mice^49^. The double-knockout mice showed impaired short-time memory accompanied by decreased accumulation of GM1 and GM3 in the brain. Furthermore, immunocytochemistry revealed that GM3 accumulated in the brains of double-knockout mice, mainly in the lysosomes of phagocytic cells, microglia, and pericytes in the brain. We observed here that the levels of *neu3s* (*neu3.1*, neu3.2, *neu3.3*, *neu3.4*, and *neu3.5*) and *neu4* in the brain of Neu1-KO zebrafish were not changed compared with those in WT^26^; however, the accumulation of GM3 might have affected the behavior of Neu1-KO zebrafish, similar to Neu3^−/−^/Neu4^−/−^ double-knockout mice. Interestingly, gangliosides have been found to not accumulate in the brain of Neu1-KO mice and patients with sialidosis^50,51^. A comparison of the composition of gangliosides in the brain between normal mice and zebrafish revealed that rich GM1 and little LacCer and GM3 were detected in the mouse brain, but not in zebrafish^51,52^. Such a difference might affect the accumulation pattern of gangliosides under a Neu1-deficiency.

In this study, we investigated the effects of Neu1 deficiency on emotional behavior in zebrafish. Since Neu1 sialidase is conserved in vertebrates, the phenotype observed in Neu1-KO zebrafish may be common in humans. However, several limitations need to be noted. First, the extent to which brain function is conserved between fish and mammals is controversial. For example, anxiety-related peptide hormones are highly conserved in vertebrates, but their expression patterns and the number of paralogous genes may not match between fish and mammals. Secondly, the distribution and structure of sialoglycans and their related enzymes in fish differ in part from that in humans. As mentioned above, the pattern of glycolipids in zebrafish also differs from that in humans. Humans possess four sialidase (Neu1, Neu2, Neu3, and Neu4), while zebrafish have six sialidases (Neu1, five Neu3 paralogs, and Neu4). To solve these limitations, it is highly desirable to elucidate the mechanism of abnormal behavior in Neu1-KO. Functional changes in central neurons caused by Neu1 deficiency should be investigated in zebrafish and mammals in comparison.

In summary, the present study revealed that Neu1 deficiency altered emotional behaviors, such as boldness and social preference, suggesting that Neu1-KO zebrafish could be used as a potential new emotional dysregulation model for the study of lysosomal desialylation. Sialoglycoconjugates involved in neuronal functions have attracted much attention, and Neu1 is the dominant sialidase in vertebrates. In addition, Neu1 is a negative regulator of lysosomal exocytosis. Therefore, Neu1-KO zebrafish might be a useful model for the study of diseases characterized by the involvement of lysosomal exocytosis.

## Materials and methods

### Fish

Neu1-KO zebrafish were established in our previous study^26^. Zebrafish larvae were kept in a 0.5 L tank and fed the paramecium, while juvenile and adult zebrafish were reared in a 3 L water aquarium and fed brine shrimp and a commercial diet (Otohime B2, Marubeni Nisshin Feed Co., Ltd, Tokyo, Japan) twice a day under a 14/10 h light and dark cycle at 28 °C until the analysis. Water quality conditions were kept as follows: pH 6.8–7.5, nitrate < 12.5 mg L^−1^, nitrite < 0.3 mg L^−1^, and chlorine < 0.1 mg L^−1^. As old Neu1-KO zebrafish (> 11 months) were shown to exhibit a decline in swimming ability due to spine curvature^26^, the present study employed young adult zebrafish (< 6 months) without symptoms of spine curvature. Mexican tetra (cavefish) were commercially obtained from a local market and kept under the same light/dark and feeding conditions as zebrafish.

All protocols for this study were approved by the Kagoshima University Committee for Animal Experiments and performed at the Faculty of Fisheries, Kagoshima University, in accordance with relevant guidelines and regulations. This study was carried out in compliance with the ARRIVE guidelines (http://www.nc3rs.org.uk/page.asp?id=1357). To minimize the effects of environmental changes to zebrafish emotional behaviors, the behavioral tests were conducted in the same room where the zebrafish breeding tank was set up.

### Fish behavioral analysis

#### Normal swimming behavior

A fish was placed at the center of the tank, measuring 20 × 18.5 × 6 cm (length × width × height). After 5 min of acclimation, their behavior was recorded for 5 min using a digital video camera (HDR-CX430, Sony, Tokyo, Japan). The camera was positioned directly above the tank. The moving locus, speed, and total distance traveled were analyzed using the Move-tr/2D software (Library, Tokyo, Japan).

#### Shoaling behavior

The shoaling behavior of zebrafish was estimated with or without acclimation, as described elsewhere^53^, with slight modifications. Briefly, 5 fish were released in the center of the transparent tank (28 × 17.5 × 14 cm), and their behaviors were immediately recorded for 5 min (without acclimation). After the test, fish were kept in the same tank. Then, 30 min after the test, the behavior of fish was recorded again for 5 min (with acclimation). The camera was fixed at the side views of the swimming tanks. The fish-internal distances between 5 fish were estimated every 30 s. The horizontal line in the middle of the tank divided the top and the bottom of the tank. Fish number in the top was counted every 5 s during the shoaling test, and the averages were calculated.

#### Aggressive behavior

Aggressive behavior was estimated as described elsewhere^54^, with slight modifications. Briefly, 2 male zebrafish were released into a transparent tank (24.5 × 7.9 × 10 cm) and their behaviors were recorded with a digital camera for 10 min. Aggressive behaviors were defined as chasing with an increase in swimming speed directed towards the opponent, circling, and biting. Swimming acceleration, swimming distance, swimming speed, and moving locus were obtained from the first 1-min behavioral data. Aggressive behavior was estimated using a 10 min analysis. The camera was fixed at the side views of the swimming tanks.

### Mirror test

These experiments were conducted according to a previous study^23^, with slight modifications. The tank was 5 cm high, 10 cm wide, and 24 cm long, and a mirror was placed on one side. A fish was placed at the center of the tank. The behavior of the fish was recorded for 15 min using a digital video camera and subsequently analyzed using the Move-tr/2D software. The camera was positioned directly above the tank. The area within 5 cm of the mirror was defined as the approach zone. The frequency and total time of invasion into the approach area, swimming distance, moving locus, and total time of chasing the opponent in the mirror were analyzed.

### Three-chambers test

The experiment was conducted as described elsewhere^55^ with slight modifications. Two small chambers (9.1 × 5.3 × 5.5 cm) were placed on one side of the experimental tank (26.8 × 19.5 × 5.5 cm). These 2 small chambers were termed empty chamber and fish chamber, respectively. The area within 1 cm of the chamber was defined as the chamber area. Then, 2 male and 2 female zebrafish were placed in the fish chamber, whereas the other one was left empty. Each test fish was placed at the center of the experimental tank. After 5 min of acclimation, the swimming behaviors of the test fish were captured for 5 min using a video camera. The camera was positioned directly above the tank. The time spent in the chamber area, invasion into each area, total distance traveled, and moving locus were analyzed using the Move-tr/2D software. For the estimation of the behavior of zebrafish toward unfamiliar fish, 4 cavefish were set in the fish tank instead of 4 zebrafish, and the following steps were performed in the same manner as before.

### Black–white preference test

The black–white preference test was conducted according to our previous study^23^ with slight modifications. The tank measured 23 × 13 × 6 cm was divided into 2 compartments (black and white background). A fish was placed in the black area of the tank, and the swimming behaviors of zebrafish were immediately captured using a video camera for 5 min. The camera was positioned directly above the tank. We measured the swimming time on the total time spent on the white side, the number of invasions into the white side, speed, and moving locus.

### Real-time PCR

The mRNA expression levels of various genes were analyzed using cDNAs obtained from zebrafish brains. The brains of Neu1-KO and WT zebrafish were dissected after anesthesia with ice water. Total RNA was extracted from the zebrafish brain using Sepasol-RNA I Super G solution (Nacalai Tesque, Kyoto, Japan), followed by cDNA synthesis using ReverTra Ace qPCR RT Master Mix with gDNA Remover (TOYOBO, Osaka, Japan).

Real-time PCR was conducted by StepOne real-time PCR (Thermo Fisher Scientific, Waltham, MA) using KOD SYBR qPCR Master Mix (TOYOBO) and specific primers for genes of *neu1*, lysosomal-associated membrane proteins 1a and 1b (*lamp1a* and *lamp1b,* respectively), cathepsin A (*ctsa*), beta-galactosidase 1 (*glb1*), N-acetylgalactosamine-6-sulfatase (*galns*), transcription factor EB (*tfeb*), isotocin (*ist*), mineralocorticoid receptor (*mr*), melanin-concentrating hormone (*mch*), arginine vasotocin (*avt*), neuropeptide Y (*npy*), orexin (*orx*), and tyrosine hydroxylase 1 and 2 (*th1* and *th2*, respectively; Supplementary Table 1). A standard curve for relative data quantification was derived from the serial dilutions of cDNA (Pffafl method). Normalization to the level of *actb* mRNA was done to compensate for the quality and quantity of mRNA in each sample.

### Lectin and western blotting

Zebrafish were anesthetized in ice water, and brains were harvested. Brains were homogenized with 300 μL of 1% Triton X-100/phosphate-buffered saline (PBS) containing 10 μg mL^−1^ leupeptin, 1 mM ethylenediaminetetraacetic acid (EDTA), and 200 μM phenylmethanesulfonyl fluoride using a BioMasher (Nippi, Tokyo, Japan), followed by centrifugation at 13,200×*g* for 15 min. The obtained lysates were resolved by SDS-PAGE and then transferred onto PVDF membranes, followed by blocking with 1% bovine serum albumin (BSA). For glycoprotein detection, the membrane was incubated with biotin-labeled MAM (Maackia amurensis lectin, recognizing Siaα2-3Gal, 1/500 diluted, J-CHEMICAL, Tokyo, Japan) and SSA lectin (Sambucus Sieboldiana Lectin, recognizing Siaα2-6Gal, 1/500 diluted, J-CHEMICAL) and then reacted with HRP-streptavidin. Accordingly, polysialic acid, lamp1, and β-actin were detected by incubation with anti-polysialic acid monoclonal antibody (1/1000 diluted, clone 735, Abnova, Taipei, Taiwan), anti-lamp1 polyclonal antibody (1/1000 diluted, ab24170, Abcam, Cambridge, England), and anti-β-actin monoclonal antibody (1/1000 diluted, 66009-1-g, Proteintech, Rosemont, IL), respectively, followed by reaction with HRP-conjugated secondary antibodies. Detection of signals was carried out using ChemiDoc Touch (Bio-Rad, Hercules, CA) with an EzWestLumi plus chemiluminescence reagent (ATTO, Tokyo, Japan). Densitometric analysis was carried out using Image Lab Touch software (Bio-Rad).

### Thin layer chromatography

Total lipids were extracted from the zebrafish brain using chloroform:methanol (1:2, 1:1, 2:1, v/v). Total lipids were fractionated into acid and neutral fractions using DEAE-Sephadex A-25 (acetate form, Sigma-Aldrich, St. Louis, MO). Each fraction was saponified with 0.2 N NaOH in methanol followed by desalting using a Sep-Pak C18 cartridge (GL Sciences, Tokyo, Japan). The obtained lipids were subjected to thin layer chromatography (TLC) plates and separated in a solvent system of chloroform/methanol/0.2% aqueous CaCl_2_ (55:45:10, v/v/v) for acid glycosphingolipids (GSLs) and chloroform/methanol/H_2_O (60:25:4, v/v/v) for neutral GSLs. The separated GSLs were visualized using orcinol-H_2_SO_4_. Densitometric analysis was carried out using Quantity One software (Bio-Rad).

### Exposure of alarm substances

Alarm substances were prepared according to Quadros et al.^56^. Briefly, the scales were removed from zebrafish by scalpel and then homogenized in cold PBS. After centrifugation at 12,000×*g*, the obtained supernatant was used as alarm substances in the study. A fish was exposed to a tank filled with 2 L of water containing alarm substances (equivalent to 0.06 fish origin/2 L) for 15 min.

### Data analysis

Group size used in this study was estimated using IBM SPSS statistics software (Armonk, NY). Results were expressed as means ± standard deviation, and all values were compared using Mann–Whitney U test.

## Supplementary Information

Supplementary Information.

## Abbreviations

GSLs: Glycosphingolipids
HPA: Hypothalamic–pituitary–adrenal
PSA: Polysialic acid
SAM: Sympathetic–adrenal–medullary
TLC: Thin layer chromatography
