# Supplementary material for: Neu1 deficiency induces abnormal emotional behavior in zebrafish
Source: Sci Rep. 2021 Jun 29;11:13477. doi: 10.1038/s41598-021-92778-9 (PMC8241872; doi:10.1038/s41598-021-92778-9)
Supplement: Supplementary file 1 — Supplementary Information. [file 41598_2021_92778_MOESM1_ESM.pdf]

Supplementary Information

Neu1 deficiency induces abnormal emotional behavior in zebrafish

Asami Ikeda [a], Mayu Komamizu [b], Akito Hayashi [b], Chiharu Yamasaki [b], Keiji Okada [b], Momoko Kawabe [a], Masaharu Komatsu [a,b] and Kazuhiro Shiozaki [a,b,*]

[a] The United Graduate School of Agricultural Sciences, Kagoshima University, Kagoshima, Japan

[b] Faculty of Fisheries, Kagoshima University, Kagoshima, Japan

*Corresponding author. 4-50-20 Shimoarata, Kagoshima 890-0056, Japan. tel/fax: +81 99 286 4170, E-mail: shiozaki@fish.kagoshima-u.ac.jp (K.Shiozaki)

Supplementary Table 1. Primers used in this study.

Supplementary Fig. 1. Polysialic acid pattern in Neu1-KO zebrafish brain. Full length blot using anti-PSA antibody shown in Fig. 7c. The region of the original blot used in main figures is shown using a red box.

Supplementary Fig. 2. Lamp1 expression patterns in Neu1-KO zebrafish brain. (a and c) Full length blots using anti-Lamp1 antibody shown in Fig. 8g and 8j, respectively. (b and d) Full length blots using anti -β actin antibody in Fig. 8g and 8j, respectively. The regions of the original blots used in main figures are shown using red boxes.

Supplementary Table 1. Primers used in this study.

Gene name	Primers	
actb	5′-CGCCATACAGAGCAGAAGCCA-3′	5′-AGCACCCTGTGCTGCTCACT-3′
mch	5′-AGGGAAACCTCGCCTTTTCG-3′	5′-AAGATCAAGGGATGGCTGGG-3′
npy	5′-AAGATGTGGATGAGCTGGGC-3′	5′-TGAATAATACTTGGCGAGCTCCT-3′
avt	5′-CGCTCTCGTCTGCCTGCTAC-3′	5′-TCTTAAGTCCCGCGCTGCTG-3′
orx	5′-TTCATGGCGCTGCTAGCTCA-3′	5′-AATTTAGCGGGCTCCTCCAGC-3′
ist	5′-TCTGGAAAGGCCTGCGGTTA-3′	5′-GCTGTTGGCCGGTTGATTGA-3′
th1	5′-TACATACGGCACGCTTCCTC-3′	5′-GAACCGCACAGAAAACGGTC-3′
th2	5′-AAAGGCTTATGGGGCTGGAC-3′	5′-GCTGCAAGTGTAGGGGTCAT-3′
mr	5′-CCCATTGAGGACCAAATCAC-3′	5′-AGTAGAGCATTTGGGCGTTG-3′
galns	5′-AACTGCCATTTTGGCCCCTA-3′	5′-TTTGGATGCCCAGACTGACC-3′
lamp1a	5′-AACCTGAGCGATGGAGACATCTT-3′	5′-CTGAGGTTTGCGCTGCTCATGTA-3′
lamp1b	5′-CAATGTCACCAATGCCAATGAT-3′	5′-CAGCGTTCCCACCAAGTAGTT-3′
glb1	5′-GCAAATGTAACCGCTGCGTT-3′	5′-AACCCAAACGCCTGATTCATTT-3′
ctsa	5′-ATGGAAGACAGCAGCATCAATT-3′	5′-GATGGTTGATGAAATGGTGTCC-3′
tfeb	5′-CCAACGACCTGGATGTACGCT-3′	5′-GCTTTCTTGCTTGATGAAGGG-3′
neu1	5′-GGTTCTGCTGAGGACAACAA-3′	5′-AGTGCATAGTACAGACCCGA-3′

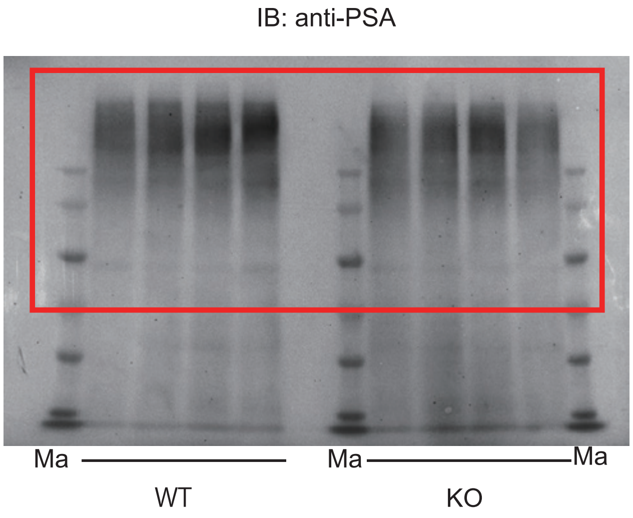

Supplementary Fig. 1

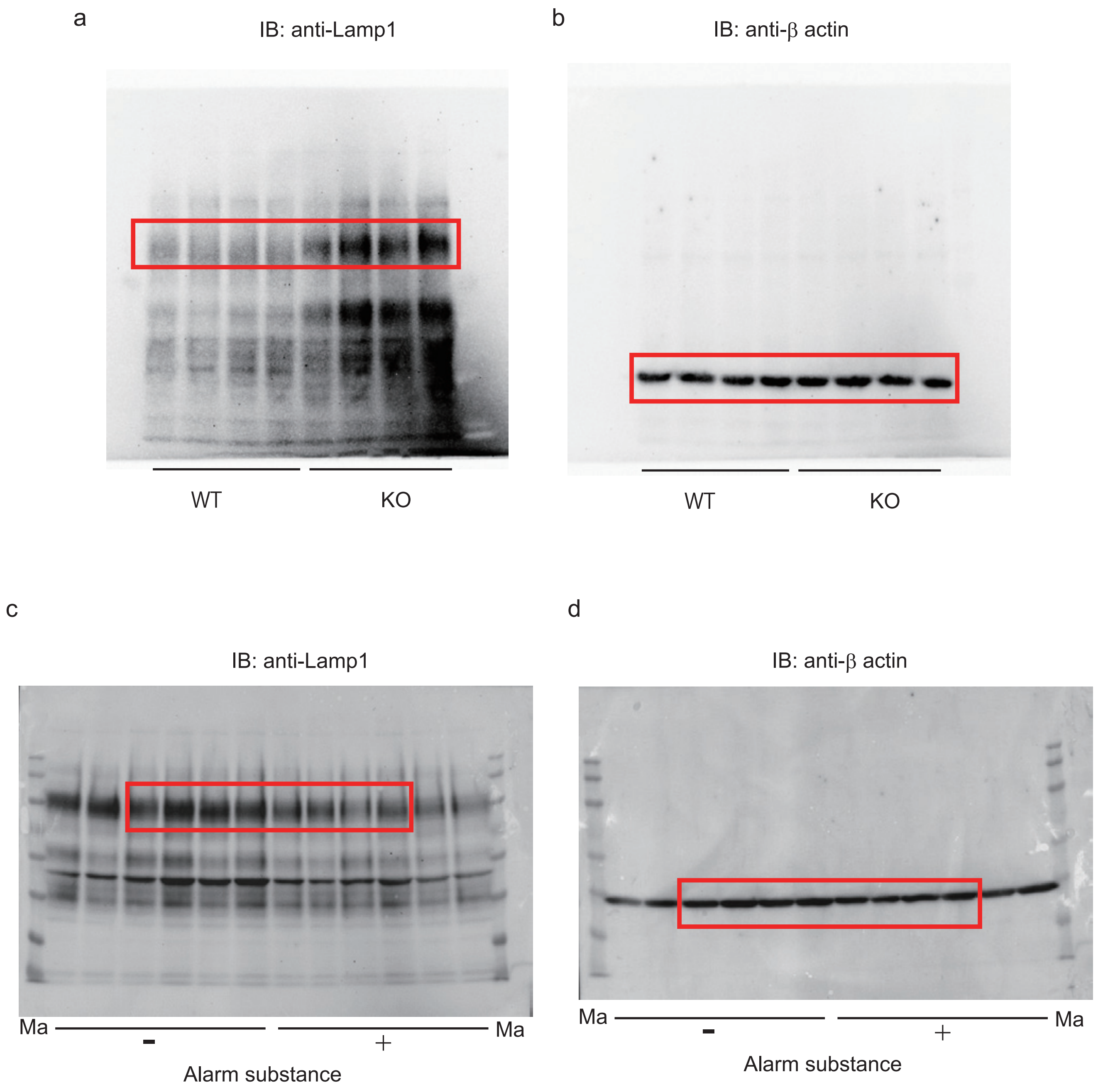

Supplementary Fig. 2
